# Prognostic Significance of Preoperative Neurological Versus Radiological Deterioration in Older Patients with Moderate-to-Mild Traumatic Brain Injury

**DOI:** 10.3390/life16010028

**Published:** 2025-12-24

**Authors:** Shin Heon Lee, Jong Tae Lee, Yong-sook Park

**Affiliations:** Department of Neurosurgery, Chung-Ang University Hospital, Chung-Ang University College of Medicine, Seoul 06973, Republic of Korea

**Keywords:** traumatic brain injury, older patients, neurological deterioration, radiological deterioration, Glasgow Coma Scale, in-hospital mortality, prognosis, surgery

## Abstract

**Background**: The prognostic value of preoperative deterioration in older patients with moderate-to-mild traumatic brain injury (TBI) remains unclear. Therefore, this study aimed to evaluate the impact of preoperative neurological and radiological deterioration on clinical outcomes in this population undergoing surgery. **Methods**: We retrospectively reviewed patients aged ≥ 65 years with moderate-to-mild TBI (Glasgow Coma Scale (GCS) ≥ 9) who underwent surgery between 2013 and 2022. Patients were grouped based on preoperative deterioration, classified as neurological (≥2-point sustained GCS drop lasting more than 1 h) or radiological (new/aggravated imaging lesions). Study outcomes included in-hospital mortality and 6-month functional status. Multivariable logistic regression was performed to identify independent predictors of outcomes. **Results**: Among 58 patients, preoperative deterioration was observed in 34 (58.6%), including 14 (24.1%) with neurological and 20 (34.5%) with radiological deterioration. In-hospital mortality was significantly higher in patients with neurological deterioration than in those without (57.1% vs. 13.6%; *p* = 0.002). Radiological deterioration alone was not associated with increased mortality or unfavorable functional outcome at 6 months. Neurological deterioration was an independent predictor of in-hospital death (adjusted odds ratio (OR), 47.9; *p* = 0.004) and unfavorable 6-month outcome (adjusted OR, 35.0; *p* = 0.014), whereas radiological deterioration was not. A lower initial GCS was also associated with unfavorable outcomes (adjusted OR, 0.5; *p* = 0.013). **Conclusions**: Preoperative neurological deterioration is an independent predictor of in-hospital mortality and unfavorable functional outcome at 6 months in older patients undergoing surgery for moderate-to-mild TBI. These findings underscore clinical neurological decline—not radiologic progression—should guide prognostication and early intervention strategies.

## 1. Introduction

Traumatic brain injury (TBI) remains a significant cause of morbidity and mortality in the older population [1,2]. The increasing incidence of moderate-to-mild TBI cases requiring neurosurgical intervention in patients aged 65 and older may be related to the aging population and increasing use of antithrombotic agents [3,4,5]. While initial neurological status and radiological findings are established prognostic indicators [6,7,8,9], the prognostic features of TBI in older adults differ from those in younger individuals [10,11].

Meanwhile, older patients presenting with moderate-to-mild TBI—defined by a Glasgow Coma Scale (GCS) score of ≥9, with mild TBI classified as GCS 13–15 and moderate TBI as GCS 9–12—are known to experience worse outcomes, including higher rates of delayed deterioration, prolonged hospitalization, and long-term disability [12,13]. Additionally, the conventional GCS-based classification may inadequately reflect clinical severity in this population, as age-related cerebral atrophy and frequent antithrombotic use can permit substantial intracranial lesion progression despite initially preserved consciousness [14,15]. Consequently, clinicoradiological dissociation may occur, and even those patients initially classified with mild TBI may require neurosurgical intervention if evolving imaging findings demonstrate significant mass effect or if neurological deterioration develops during observation [16]. Overall, the need for surgery in older patients is often determined by the dynamic neurological and radiological course rather than by initial injury severity alone.

Neurological or radiological deterioration during the preoperative period may reflect ongoing secondary injury processes such as hematoma expansion, ischemia, or evolving cerebral edema [17,18,19]. Although prior studies have highlighted the prognostic relevance of early neurological decline and radiological progression in TBI [20,21], the specific impact of these metrics in older patients with moderate-to-mild TBI—particularly those undergoing surgical intervention—remains insufficiently defined. Moreover, the clinical significance of preoperative deterioration—both neurological and radiological—remains underexplored in patients who eventually undergo surgery. Therefore, differentiating between neurological and radiological deterioration may provide further insights into prognostication and surgical decision-making.

Thus, this study aimed to evaluate the impact of preoperative neurological and radiological deterioration on in-hospital mortality and 6-month functional outcome in older patients aged 65 years or older undergoing surgical intervention for moderate-to-mild TBI. The age threshold of 65 years was selected, as this age is commonly used to define a clinically distinct older population [22]. Hence, by distinguishing between neurological and radiological deterioration before surgery, we sought to clarify the independent prognostic relevance of the type of deterioration in this vulnerable group.

## 2. Materials and Methods

### 2.1. Study Design and Patient Selection

We retrospectively evaluated a cohort of older patients (aged ≥ 65 years) who underwent neurosurgical intervention for moderate-to-mild TBI at our tertiary referral center between January 2013 and December 2022. Moderate-to-mild TBI was defined as an initial GCS score of 9 or higher upon presentation, as the GCS enables rapid, standardized, and repeated assessment of consciousness and facilitates detection of dynamic neurological deterioration in the acute preoperative period [23]. A total of 58 patients met the inclusion criteria and were included in the final analysis.

Patients were excluded upon meeting any of the following criteria: (1) initial GCS score < 9; (2) death within 72 h of admission; (3) surgical evacuation for epidural hematoma or chronic subdural hematoma; (4) presence of severe concomitant systemic injuries (e.g., major thoracoabdominal trauma requiring surgical intervention or causing hemodynamic instability). Patients who died within 72 h of admission were excluded to focus on perioperative prognostic factors among surgically managed patients and to reduce confounding from early deaths attributable to overwhelming primary injury or non-neurological causes before surgery. Only patients who underwent surgery for acute traumatic intracranial lesions such as subdural hematoma, intracerebral hemorrhage, or contusional hemorrhage were included.

This study was approved by the appropriate Institutional Review Board (IRB No. 2505-007-19573, approval date: 15 May 2025) and was conducted in accordance with the principles of the Declaration of Helsinki. The requirement for informed consent was waived due to the retrospective nature of the study.

### 2.2. Neurological and Radiological Monitoring Protocol

Neurological status was continuously assessed using the GCS at hourly intervals from the time of admission to the emergency department. This hourly monitoring protocol was maintained in the intensive care unit and step-down wards by trained nursing staff.

Follow-up brain imaging was performed in accordance with institutional protocol. High-energy trauma was defined as injury mechanisms involving substantial kinetic force, such as motor vehicle collisions or falls from height, whereas low-energy trauma referred to ground-level falls or minor blunt impacts. For patients with high-energy trauma or those receiving antithrombotic therapy, an initial follow-up scan was routinely obtained within 3–6 h after the first emergency imaging, and another scan was performed the following day. In patients with lower-energy trauma who were not receiving antithrombotic therapy, follow-up imaging was conducted on the day after admission. Additional imaging was scheduled for all patients on postoperative days 5–7, or earlier if specific clinical changes, such as new neurological deficits or unexplained neurological deterioration, were observed. This imaging protocol was applied as standard institutional practice throughout the study period. Minor variations in imaging timing occurred in a limited number of cases due to clinical circumstances; however, all patients underwent sufficient preoperative follow-up imaging to evaluate the radiological deterioration. No systematic deviations from the institutional imaging protocol were identified.

### 2.3. Definition of Preoperative Deterioration

Preoperative deterioration was categorized as neurological or radiological based on clinical examination and neuroimaging findings obtained between emergency department admission and surgery. This definition encompassed both deterioration that occurred during the observation period before the surgical decision and after the decision for surgery, but before the operation. Neurological deterioration was defined as a sustained decrease of ≥2 points in the GCS persisting for more than 1 h, as observed between any two consecutive neurological evaluations, irrespective of setting, including the emergency department, intensive care unit, or ward. Radiological deterioration was defined as either radiographical progression of pre-existing lesions (e.g., hematoma expansion or increased midline shift) or the emergence of new intracranial abnormalities (e.g., new hematoma formation, hydrocephalus, or ischemic lesions) on follow-up computed tomography (CT) or magnetic resonance imaging (MRI) performed before surgery.

Based on these criteria, patients were categorized into three groups according to preoperative deterioration status: no deterioration (*n* = 24), neurologic deterioration (*n* = 14), and radiologic deterioration (*n* = 20). In cases where both neurological and radiological deteriorations were observed, patients were classified into the neurological deterioration group, as neurological deterioration represents a clinically overt manifestation, thereby ensuring mutually exclusive group classification.

### 2.4. Data Collection

For each patient, we retrospectively collected demographic and clinical data, including age, sex, comorbid conditions (hypertension, diabetes mellitus, cardiac disease, and prior cerebrovascular events), and pre-injury use of antithrombotic agents. Detailed radiological findings on initial brain CT scans were reviewed, including the presence of subdural hemorrhage, intracerebral hemorrhage, subarachnoid hemorrhage, and the Rotterdam CT score. The mechanism of injury was categorized as either high-energy or low-energy. The timing of deterioration was documented for each patient. Information regarding the type of surgical procedure—craniotomy or craniectomy—was also recorded. All data were obtained through a comprehensive review of electronic medical records and imaging archives and were independently verified by two investigators to ensure consistency and accuracy.

### 2.5. Outcome Measures

The primary outcomes of this study were in-hospital mortality and functional outcome at 6 months following surgery. Functional outcome was assessed using the Glasgow Outcome Scale (GOS) and was dichotomized into favorable (GOS 4–5) and unfavorable (GOS 1–3) categories for analysis. The 6-month time point was selected as it is generally considered to represent a relatively stable phase of neurologic recovery [24], while limiting the influence of aging-related functional decline, comorbidities, and intercurrent events that may confound longer-term outcome assessment in older patients [25]. GOS scores were determined through outpatient clinic follow-up by the clinical staff.

### 2.6. Statistical Analysis

Statistical analyses were performed using Python software (version 3.8.10; Python Software Foundation, Wilmington, DE, USA). Continuous variables are presented as the mean ± standard deviation or median with interquartile range (IQR), depending on data distribution; categorical variables are expressed as frequencies and percentages. Normality was assessed using the Shapiro–Wilk test, and homogeneity of variances was evaluated using Levene’s test; parametric or nonparametric tests were applied accordingly. Between-group comparisons of continuous variables were performed using Student’s t-test or the Mann–Whitney U test, and categorical variables were compared using the chi-square test or Fisher’s exact test, as appropriate. Multivariable logistic regression analyses were conducted to identify independent predictors of in-hospital mortality and unfavorable functional outcome at 6 months, with variables selected a priori based on clinical relevance, and adjusted odds ratios (ORs) with 95% confidence intervals (CIs) were reported. Multicollinearity was assessed using variance inflation factors, and model calibration was evaluated using the Hosmer–Lemeshow goodness-of-fit test. A two-tailed *p*-value of <0.05 was considered statistically significant.

## 3. Results

### 3.1. Patient Characteristics

We analyzed data for 58 older patients with moderate-to-mild TBI who underwent surgical intervention. Among these patients, 34 (58.6%) experienced preoperative neurological and/or radiological deterioration. The mean age was 76.4 ± 6.6 years; 38 patients (65.5%) were male and 20 (34.5%) were female. Baseline characteristics, including age, sex, comorbidities (hypertension, diabetes, cardiac disease, prior stroke), and antithrombotic use, did not significantly differ between patients with and without deterioration (Table 1).

Subdural hemorrhage was the most common radiological finding (75.9%), followed by subarachnoid hemorrhage (39.7%) and intracerebral hemorrhage (24.1%). Subarachnoid hemorrhage was more prevalent in patients with deterioration than in patients without deterioration (50.0% vs. 25.0%), showing a trend toward significance (*p* = 0.064). The median Rotterdam CT score for the entire cohort was 3.0 (IQR, 2.0–4.0), and did not significantly differ between patients with and without deterioration (3.0 (IQR, 2.0–4.0) vs. 2.0 (IQR, 2.0–3.0); *p* = 0.176), indicating comparable baseline radiological severity.

High-energy trauma accounted for 13.8% with no significant intergroup difference between patients with and without deterioration (11.8% vs. 16.7%; *p* = 0.706). Similarly, the initial GCS scores were comparable between patients with and without deterioration (12.0 (IQR, 10.0–14.0) vs. 13.0 (IQR, 12.0–15.0); *p* = 0.220). Surgical procedures included craniectomy in 36.2% and craniotomy in 63.8% of cases, with no significant difference in surgical modality between patients with and without deterioration (*p* = 0.414).

Notably, in-hospital mortality was significantly higher among patients with preoperative deterioration (35.3%) than in those without (8.3%; *p* = 0.028). However, the proportion of patients with unfavorable functional outcomes at 6 months (GOS ≤ 3) was similar in both groups (67.6% vs. 62.5%; *p* = 0.777). The median interval between hospital arrival and the onset of deterioration (neurological and/or radiological) was 11.9 h (IQR, 5.1–38.0 h). The majority of preoperative deterioration cases (25 of 34, 73.5%) occurred within 24 h of admission, with more than half (18 of 34, 52.9%) developing within the first 12 h.

### 3.2. Clinical Outcomes According to Deterioration Type

Among the 34 patients with deterioration, 14 (41.2%) exhibited neurological deterioration, while 20 (58.8%) showed radiological deterioration. The outcomes in these subgroups were further analyzed (Table 2).

When stratified by deterioration type, patients with neurological deterioration showed a significantly increased in-hospital mortality rate compared to those without (57.1% vs. 13.6%; *p* = 0.002). Similarly, an unfavorable outcome at 6 months (GOS ≤ 3) was more frequent in patients with neurological deterioration than in those without (85.7% vs. 54.5%), although this difference did not reach statistical significance (*p* = 0.057). Radiological deterioration alone was not significantly associated with either in-hospital mortality (*p* = 0.751) or an unfavorable outcome at 6 months (*p* = 0.570).

**Figure 1:**
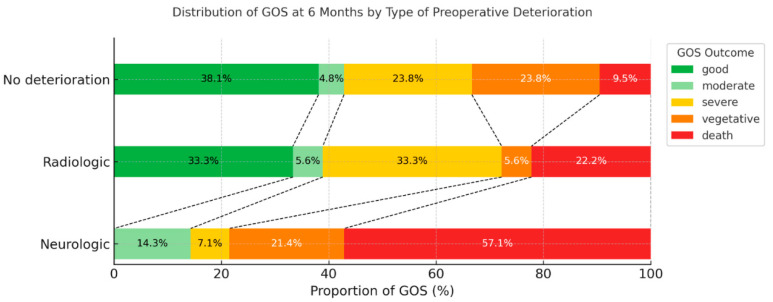
Distribution of 6-month Glasgow Outcome Scale (GOS) by type of preoperative deterioration. Stacked horizontal bar chart of the distribution of 6-month GOS categories according to preoperative deterioration type. Functional outcomes are displayed as proportions within each group.

The distribution of the GOS scores at 6 months revealed that none of the patients with neurological deterioration achieved a good recovery (GOS 5), with a higher proportion of mortality or severe disability cases (Figure 1).

### 3.3. Multivariable Analyses for Clinical Outcomes

**Figure 2:**
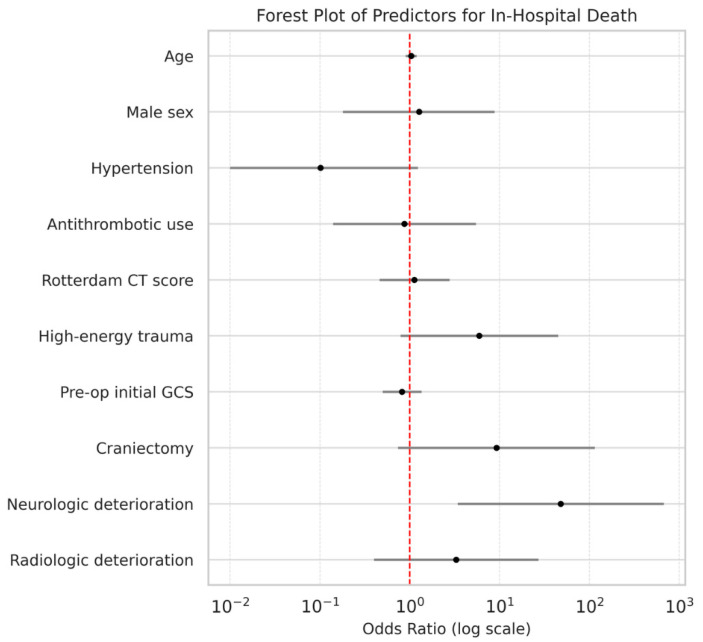
Forest plot of predictors for in-hospital mortality. Adjusted ORs with 95% CIs from multivariable logistic regression are shown. ORs are plotted on a logarithmic scale; a two-tailed *p* < 0.05 was considered statistically significant.

In multivariable logistic regression for in-hospital mortality (Table 3; Figure 2), neurologic deterioration emerged as an independent predictor (adjusted OR, 47.9; 95% CI, 3.4–670.9; *p* = 0.004). Other variables, including age, sex, hypertension, antithrombotic use, Rotterdam CT score, high-energy trauma, initial GCS, and surgical procedure (craniectomy vs. craniotomy), were not independently associated with in-hospital mortality.

**Figure 3:**
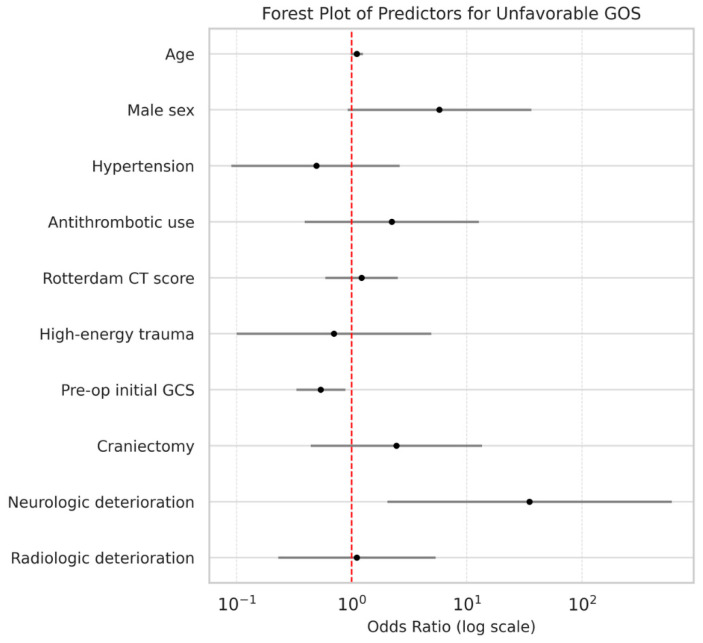
Forest plot of predictors for unfavorable functional outcome (GOS ≤ 3) at 6 months. Adjusted ORs with 95% CIs from multivariable logistic regression are shown. ORs are plotted on a logarithmic scale; a two-tailed *p* < 0.05 was considered statistically significant.

In multivariable logistic regression for unfavorable outcome at 6 months (Table 4; Figure 3), neurological deterioration (adjusted OR, 35.0; 95% CI, 2.0–601.7; *p* = 0.014) and lower preoperative GCS (adjusted OR, 0.5; 95% CI, 0.3–0.9; *p* = 0.013) were independently associated with worse outcomes. Radiological deterioration did not show significant predictive value for either outcome measure.

## 4. Discussion

This study demonstrates that preoperative neurologic deterioration is an independent predictor of both in-hospital mortality and unfavorable functional outcome at 6 months in older patients undergoing surgery for moderate-to-mild TBI. In contrast, radiological deterioration without neurological decline was not significantly associated with worse outcomes. These findings suggest that dynamic neurological decline before surgery may serve as a clinically relevant marker of secondary injury progression in this population, even when the initial GCS is relatively preserved.

Previous studies have reported that early neurological decline is associated with poor prognosis in patients with severe TBI [26,27]; however, data focusing specifically on moderate-to-mild TBI in the older surgical population remains limited. Reynolds et al. (2003) highlighted that older patients undergoing anticoagulation therapy may experience rapid neurological deterioration—often within 6 h of injury—despite having an initially normal neurological status [28]. Additionally, a meta-analysis by Marincowitz et al. (2018) examined broader or more heterogeneous TBI populations, estimating an 11.7% risk of clinical deterioration in patients with mild TBI and CT abnormalities [29]. However, the study did not differentiate between types of deterioration and did not focus on older individuals. Our findings build upon and refine these earlier observations by categorizing deterioration based on the type and focusing specifically on a cohort of older individuals undergoing surgery for moderate-to-mild injury. We found that neurological deterioration occurred in 24.1% of patients and was associated with worse mortality and functional outcomes, highlighting the potential prognostic significance of neurological decline.

Lower preoperative GCS scores were independently associated with unfavorable functional outcome at 6 months, reaffirming the prognostic importance of baseline neurological status, as demonstrated in prior studies [30,31]. However, our findings indicate that dynamic neurological deterioration, rather than static GCS measurements alone, provides additional prognostic information. In addition, when comparing results by deterioration status, we found that the initial GCS was similar across deterioration groups, suggesting that the initial GCS was not predictive of deterioration. Although alternative instruments such as the Functional Status Examination or the Disability Rating Scale assess broader disability, these metrics are less suitable for frequent acute bedside monitoring; thus, the use of the GCS reflects our focus on early, dynamic neurological deterioration rather than long-term functional impairment.

Interestingly, isolated radiological progression was not associated with worse outcomes, despite being present in more than one-third of patients. Several pathophysiological and clinical factors may explain the limited prognostic value of radiological deterioration in older patients with TBI; however, these explanations should be regarded as hypothesis-generating rather than definitive. Radiological assessment primarily reflects structural changes, such as hematoma expansion or midline shift, and may not adequately capture dynamic physiologic processes directly related to the neurological outcome, including intracranial pressure dynamics, cerebral perfusion, and metabolic dysfunction. In older adults, age-related cerebral atrophy may further attenuate the association between radiological progression and clinical deterioration by permitting lesion expansion without a proportional increase in intracranial pressure or immediate neurological decline [32]. Consequently, radiological deterioration may represent compensated processes that remain clinically tolerated, whereas neurological deterioration more directly reflects failure of compensatory mechanisms. Prior studies also caution against over-reliance on imaging alone in older patients, especially those with preserved neurological function [16,33].

From a clinical standpoint, our findings emphasize the importance of early and repeated neurological assessments in older patients with TBI, particularly in the preoperative period. Neurological deterioration, even in the absence of radiographic progression, should prompt urgent neurosurgical evaluation, as this decline may represent a critical inflection point in the clinical course of the patient. Reliance on imaging alone may underestimate the extent of the evolving injury, especially in cases with dissociation between the radiological and clinical findings [34]. Conversely, radiological progression without neurological decline warrants more individualized interpretation, particularly in patients with significant reserve capacity from brain atrophy [35]. These results suggest a neurological-centered conceptual framework for older patients with TBI in which dynamic neurological deterioration guides surgical decision-making and subsequent treatment strategies, with radiological findings serving a supportive rather than primary prognostic role.

This study has several limitations. First, our retrospective single-center design may limit generalizability, although findings remained robust after multivariable adjustment. In addition, the relatively small cohort size (*n* = 58) and imbalance in participant numbers across subgroups may increase statistical uncertainty and the risk of type I error; therefore, the observed associations should be interpreted with caution rather than as definitive. Second, although the definition of deterioration was based on clinical criteria, variability in documentation practices may have introduced inconsistencies in the associated application. Furthermore, our operational definition of radiological deterioration primarily captures acute morphological changes in the brain and may not fully account for delayed secondary injury mechanisms, such as vasospasm. Third, hierarchical classification of patients with combined neurological and radiological deterioration into the neurological group may have attenuated the apparent prognostic effect of isolated radiological deterioration; therefore, the results should be interpreted conservatively.

A further conceptual limitation lies in the decision to restrict the study to patients undergoing surgery. This study aimed to identify prognostic factors among surgically managed older patients with moderate-to-mild TBI; consequently, patients with severe TBI (GCS < 9) or those managed non-surgically were excluded, which limits applicability across the full spectrum of TBI severity and management strategies. Moreover, patients who died within 72 h of admission were excluded to focus on perioperative prognostic factors, which may have introduced survivorship bias, particularly in the mortality analyses.

Despite these limitations, our findings provide preliminary insights that contribute to a more nuanced understanding of TBI in older adults, with a focus on moderate-to-mild injury severity. Specifically, we advocate for evaluating neurological status in addition to relying on radiological findings to guide clinical decision-making and prognostication in older patients undergoing surgical management for moderate-to-mild TBI. Nonetheless, future multicenter prospective studies are warranted to validate and extend these observations.

## 5. Conclusions

Neurological deterioration is a more important prognostic indicator in older patients undergoing surgery for moderate-to-mild TBI than radiological progression alone. These findings highlight the critical importance of vigilant and continuous neurological assessment in this high-risk population, even when initial GCS scores appear preserved.

## Figures and Tables

**Table 1:** Baseline characteristics of patients with and without preoperative deterioration.

Variable	Total (*n* = 58)	No Deterioration (*n* = 24)	Deterioration (*n* = 34)	*p*-Value
Age	76.4 ± 6.6	76.1 ± 6.8	76.7 ± 6.5	0.750
Male sex	38 (65.5)	17 (70.8)	21 (61.8)	0.579
Hypertension	34 (58.6)	13 (54.2)	21 (61.8)	0.598
Diabetes mellitus	16 (27.6)	6 (25.0)	10 (29.4)	0.773
Cardiac disease	7 (12.1)	1 (4.2)	6 (17.6)	0.221
Prior stroke	8 (13.8)	1 (4.2)	7 (20.6)	0.123
Antithrombotic use	17 (29.3)	6 (25.0)	11 (32.4)	0.575
Initial radiological findings				
Subdural hemorrhage	44 (75.9)	21 (87.5)	23 (67.6)	0.121
Intracerebral hemorrhage	14 (24.1)	6 (25.0)	8 (23.5)	1.000
Subarachnoid hemorrhage	23 (39.7)	6 (25.0)	17 (50.0)	0.064
Rotterdam CT score	3.0 (2.0–4.0)	3.0 (2.0–4.0)	2.0 (2.0–3.0)	0.176
High-energy trauma	8 (13.8)	4 (16.7)	4 (11.8)	0.706
Preoperative initial GCS	13.0 (11.0–14.8)	12.0 (10.0–14.0)	13.0 (12.0–15.0)	0.220
Surgical procedure				0.414
Craniectomy	21 (36.2)	7 (29.2)	14 (41.2)	
Craniotomy	37 (63.8)	17 (70.8)	20 (58.8)	
In-hospital death	14 (24.1)	2 (8.3)	12 (35.3)	0.028
Unfavorable GOS at 6 months	38 (65.5)	15 (62.5)	23 (67.6)	0.777
Time interval to deterioration, h			11.9 (5.1–38.0)	
Deterioration ≤ 24 h			25 (73.5)	
Deterioration ≤ 12 h			18 (52.9)	

Data are presented as the mean ± standard deviation, median (IQR), or proportion (%). Continuous variables were compared using Student’s *t*-test or the Mann–Whitney U test, and categorical variables using the chi-square test or Fisher’s exact test, as appropriate; a two-tailed *p* < 0.05 was considered statistically significant. GCS, Glasgow Coma Scale; GOS, Glasgow Outcome Scale.

**Table 2:** Association between deterioration type and clinical outcomes.

Outcome	Neurological Deterioration		*p*-Value	Radiological Deterioration		*p*-Value
	No (*n* = 44)	Yes (*n* = 14)		No (*n* = 38)	Yes (*n* = 20)	
In-hospital death	6 (13.6)	8 (57.1)	0.002	10 (26.3)	4 (20.0)	0.751
Unfavorable GOS	24 (54.5)	12 (85.7)	0.057	25 (65.8)	11 (55.0)	0.570

Among patients with preoperative deterioration, outcomes were compared according to deterioration type (neurological vs. radiological). Outcomes are presented as proportions (%) and were compared using the chi-square test or Fisher’s exact test, as appropriate; a two-tailed *p*-value < 0.05 was considered statistically significant. GOS, Glasgow Outcome Scale.

**Table 3:** Multivariable logistic regression for in-hospital mortality.

Outcome	Variable	β	Adjusted OR (95% CI)	*p*-Value
In-hospital death	Age	0.04	1.0 (0.9–1.2)	0.623
	Male sex	0.24	1.3 (0.2–8.8)	0.806
	Hypertension	−2.29	0.1 (0.0–1.2)	0.072
	Antithrombotic use	−0.14	0.9 (0.1–5.5)	0.884
	Rotterdam CT score	0.12	1.1 (0.5–2.8)	0.797
	High-energy trauma	1.78	5.9 (0.8–45.0)	0.084
	Preoperative initial GCS	−0.20	0.8 (0.5–1.4)	0.434
	Craniectomy	2.22	9.2 (0.7–115.0)	0.084
	Neurological deterioration	3.87	47.9 (3.4–670.9)	0.004
	Radiological deterioration	1.19	3.3 (0.4–27.1)	0.269

A two-tailed *p* < 0.05 was considered statistically significant. GCS, Glasgow Coma Scale; OR, odds ratio; CI, confidence interval.

**Table 4:** Multivariable logistic regression for unfavorable outcome.

Outcome	Variable	β	Adjusted OR (95% CI)	*p*-Value
Unfavorable GOS	Age	0.10	1.1 (1.0–1.3)	0.092
	Male sex	1.75	5.8 (0.9–36.3)	0.061
	Hypertension	−0.70	0.5 (0.1–2.6)	0.406
	Antithrombotic use	0.80	2.2 (0.4–12.7)	0.367
	Rotterdam CT score	0.20	1.2 (0.6–2.5)	0.591
	High-energy trauma	−0.36	0.7 (0.1–4.9)	0.721
	Preoperative initial GCS	−0.62	0.5 (0.3–0.9)	0.013
	Craniectomy	0.90	2.4 (0.4–13.6)	0.305
	Neurological deterioration	3.56	35.0 (2.0–601.7)	0.014
	Radiological deterioration	0.10	1.1 (0.2–5.3)	0.901

A two-tailed *p* < 0.05 was considered statistically significant. GCS, Glasgow Coma Scale; GOS, Glasgow Outcome Scale; OR, odds ratio; CI, confidence interval.

## Data Availability

The data presented in this study are available from the corresponding author upon request due to privacy concerns.

## Abbreviations

TBI	traumatic brain injury
GCS	Glasgow Coma Scale
CT	computed tomography
MRI	magnetic resonance imaging
GOS	Glasgow Outcome Scale
CI	confidence interval
OR	odds ratio
SD	standard deviation
